# 

**DOI:** 10.1192/bjb.2024.36

**Published:** 2024-10

**Authors:** Hannah Kay Ali

**Affiliations:** Consultant psychiatrist with East London NHS Foundation Trust, London, UK. Email: hkali@doctors.org.uk

**Figure:**
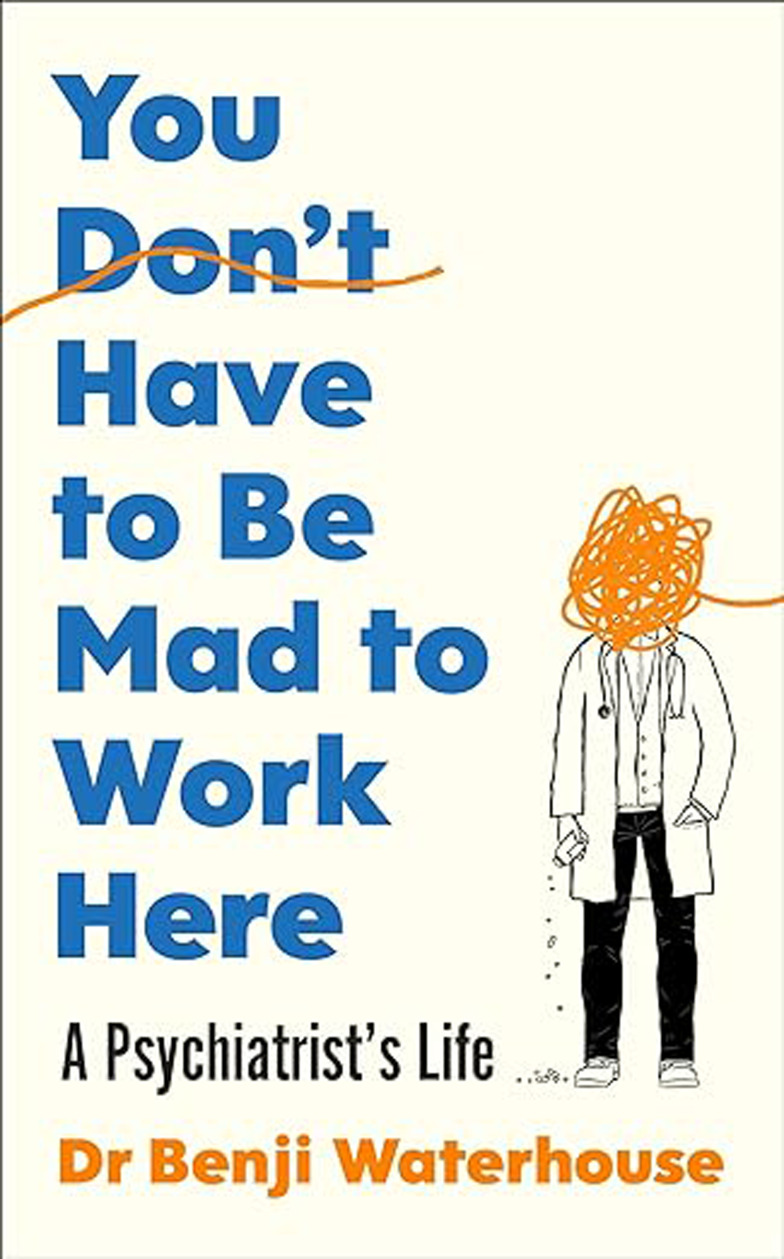

Dr Benji Waterhouse's debut book offers an engaging blend of humour and insight, catering primarily to the general public while holding significant value for psychiatrists. Through his witty and self-aware narrative, comedian Waterhouse invites readers into the tumultuous world of a trainee psychiatrist, where personal and professional challenges intertwine.

Drawing parallels with acclaimed works like Adam Kay's *This is Going to Hurt* and James Herriot's *All Creatures Great and Small*, Waterhouse's storytelling resonates with its candid exploration of the profession's nuances. Despite the comedic lens, he skilfully navigates complex topics such as the Mental Health Act, medication and psychiatry's portrayal in popular culture, sparking introspection among readers.

One might assume that comedy could trivialise such weighty subjects, but Waterhouse's approach proves otherwise. With sensitivity and depth, he sheds light on the darkest corners of psychiatry, offering poignant reflections on patient care, systemic flaws and the toll of the nature of this work. He makes use of common cases that he has encountered, and his own mental health and personal experiences, to explore these themes in an engaging way.

Beyond the humour lies a narrative that strikes a chord with psychiatrists, encapsulating the unique challenges we face. Be it discussions on breakaway training, fear of the coroner's court, burnout or CASC exams – his storytelling not only captures the challenges we face daily but also celebrates the resilience and camaraderie that sustain us.

On the surface, it's a funny and entertaining read. But in exploring the highs and lows of his journey, Waterhouse also reminds us why we chose this path and reignites our passion for making a difference in the lives of others. His book serves as both a mirror reflecting our shared experiences and a beacon of hope guiding us through the darkest moments of our profession.

